# Factors Associated With Healthcare Workers' Insomnia Symptoms and Fatigue in the Fight Against COVID-19, and the Role of Organizational Support

**DOI:** 10.3389/fpsyt.2021.652717

**Published:** 2021-03-26

**Authors:** Xia Zou, Shaokun Liu, Jie Li, Wen Chen, Jiali Ye, Yuan Yang, Fenfen Zhou, Li Ling

**Affiliations:** ^1^Global Health Research Center, Guangdong Provincial People's Hospital, Guangdong Academy of Medical Sciences, Guangzhou, China; ^2^Department of Information, Sun Yat-sen University Cancer Center, Sun Yat-sen University, Guangzhou, China; ^3^Department of Medical Statistics, School of Public Health, Sun Yat-sen University, Guangzhou, China

**Keywords:** COVID-19, healthcare workers, insomnia, fatigue, organizational support

## Abstract

**Background:** Healthcare workers (HCWs) have been exposed to increased risks of insomnia and fatigue during the COVID-19 pandemic. In this study, we identify important risk factors associated with insomnia symptoms and fatigue among HCWs, and evaluate the effect of organizational support on insomnia and fatigue symptoms.

**Methods:** This is an online cross-sectional survey of HCWs in China administered during the COVID-19 epidemic (from February 27, 2020 to March 12, 2020). We employed the AIS-8 scale for insomnia screening, and a self-reported ten-point scale to evaluate subjects' degrees of fatigue. We also designed a four-point scale to assess the degree of social support provided on an organizational level. Additionally, we conducted logistic regression analysis to identify risk factors.

**Results:** This study included a total of 3,557 participants, 41% of which consisted of non-frontline HCWs and 59% of which was frontline HCWs. Of the non-frontline HCWs, 49% reported insomnia symptoms, and 53.8% reported a moderate to high degree of fatigue. Meanwhile, among the frontline HCWs, the percentages for insomnia and moderate to high fatigue were 63.4% and 72.2%, respectively. Additionally, frontline HCWs and HCWs employed at Centers for Disease Control and Prevention (CDCs) had elevated risks of insomnia and fatigue. However, with increased organizational support, insomnia symptoms decreased among frontline HCWs. Also, organizational support mitigated the positive correlation between daily working hours and degree of fatigue among HCWs.

**Conclusion:** Frontline HCWs and staff in Chinese CDCs have been at a high risk of insomnia symptoms and fatigue during the fight against COVID-19. This study provides evidence for the positive effects of organizational support in relation to insomnia and fatigue among HCWs. This sheds light on government responses to the COVID-19 epidemic for other countries.

## Introduction

The 2019 coronavirus (COVID-19) pandemic has been characterized by high transmissibility. As of April 3, 2020, it has caused 9.76 million infections and 50,414 deaths worldwide (1). To contain the epidemic within its borders, the Chinese government has declared the highest level of public health emergency alert, and has taken rapid and comprehensive action to limit its spread. This has included enacting strict quarantine measures, improving case identification, patient diagnosis, treatment, and psychological interventions, and improving the training of healthcare workers (HCWs), as well as strengthening logistical support and establishing units and hospitals for quarantined patients (2–5). Nationwide, these policies have resulted in millions of clinical staff, public health workers and other HCWs working consecutive days on the front lines during this period (6).

Front line HCWs have faced tremendous challenges during the COVID-19 epidemic. This has included an ever-increasing suspected and confirmed COVID-19 caseload, excessive workloads, isolation from friends and families, feelings of inadequate support, and discrimination (7). In such an unprecedented stressful situation, insomnia, feelings of fatigue, and even burn-out have been common. Insomnia has been the earliest and most prominent symptom reported by patients coping with stress (8), and fatigue has been the most common and persistent symptom caused by insomnia (9). These symptoms can result in daytime exhaustion, medical and psychiatric disorders, and lowered immune response among HCWs. Consequently, this elevates their risk of infection, and even death (10–12). Although only a few studies have reported data concerning insomnia and degree of fatigue among HCWs during the COVID-19 epidemic, these studies have identified several putative factors associated with both of these ailments.

For example, it has been documented that high levels of social support attenuate insomnia and fatigue symptoms associated with stress (13–15). In particular, previous studies have reported that organizational support improves job satisfaction for HCWs with high burnout levels (16). To help front line HCWs combat the challenges of this stressful situation, the Chinese government has launched a series of measures designed to support HCWs and their families. These measures have included providing protective equipment and training, improving subsidies, offering incentives, guaranteeing adequate daily necessities for HCWs and their families, shifting work schedules and providing psychological interventions (2). However, to date, no studies have examined the effect of organizational support on insomnia and fatigue among HCWs during the COVID-19 epidemic.

In addition, work-related factors and mental factors were also reported to be associated with insomnia and fatigue. For example, previous study shows that doctors whose inter-shift interval <10 h were more likely to be sleepless and fatigued (17). In Leblanc's study, psychological factors (include depression and anxiety) were found to the most important risk factors of new onset insomnia (18). Williamson et al. reported a negative association between fatigue and mental health measures (19). But the factors associated with insomnia and fatigue of HCWs in during the COVID-19 epidemic have not been well-understood.

In this study, we identify the factors associated with insomnia and fatigue among HCWs, and evaluate organizational support's effect on insomnia and fatigue in HCWs. To this end, we conducted an online cross-sectional survey during the COVID-19 epidemic. This report may be helpful for other countries dealing with the psychological problems and fatigue that HCWs face in the fight against COVID-19.

## Methods

### Study Design and Participants

We conducted an online cross-sectional survey targeting HCWs in China during the early stages of the COVID-19 epidemic (February 27, 2020 to March 12, 2020).

Participants were eligible if they: (1) were engaged in work related to healthcare, including, but not limited to, clinical doctors, nurses, medical laboratory staff, public health practitioners, health management personnel and healthcare research staff; and (2) were able to provide written informed consent. Those who were unable to complete the survey were excluded from participation.

The questionnaire was designed and piloted among HCWs before the online survey was deployed. A brief questionnaire which can be finished within 10 min was finalized to improve the acceptance of the survey. We employed a popular electronic survey tool (*Wenjuanxing*, Changsha Ranxing Information Technology Co. Ltd, China) to generate a link to the online questionnaire. Participants were recruited through peer referral. The questionnaire link was disseminated via WeChat, a popular social media platform in which users register with a unique phone number. We performed online written informed consent before the survey to ask whether participants would like to participate. It included the aims, contents, risks and benefits of participating in this study. If they answered “yes,” the survey would begin. Otherwise, the survey was terminated. Once a participant submitted the questionnaire, he or she would not be able to access it again.

### Ethical Approval

This study has been approved by the ethical committee at Sun Yat-sen University [(2020) No. 011].

### Measures

#### Insomnia and Fatigue (Dependent Variables)

We used the Athens Insomnia Scale-8 (AIS-8) to assess risk of insomnia. This instrument was developed in 1985 based on the International Classification of Diseases-10 criteria, and it has been used in many evaluations of insomnia severity (20–22). The scale contains eight items which were coded on a scale from 0 to 3 (0 = none, 1 = mild, 2 = significant, 3 = severe). A cut-off point of six was used to identify participants who had insomnia. Previous studies have demonstrated this scale's reliability and validity (23). Accordingly, the instrument in this study demonstrated good internal consistency (Cronbach's α = 0·89).

Participants were asked to evaluate their degree of fatigue during the previous week. We used a brief continuous numerical scale ranging from 0 to 10 for evaluation (0 = no fatigue, 10 = burn out).

#### Independent Variables

Participants' demographic information was collected, including sex, age, educational attainment, marital status, occupation(s), job title(s) and employer. Participants were also asked to describe their role in the COVID-19 response effort (1 = front line healthcare worker, 2 = non-front line healthcare worker). Front line HCWs were defined as those directly engaged in work related to the detection, testing, diagnosis and treatment of COVID-19 patients.

Data were also collected regarding work-related factors, including daily working hours, shift length and hours of sleep *per day*.

Participants were asked the extent to which they perceived support from organizations (this included government offices, state-owned enterprises, and private companies) and individuals (including friends, colleagues and their families). We designed a four-point scale to measure perceived degree of social support. Each grade was coded on a scale from 0 to 3 (0 = not at all, 1 = low, 2 = moderate, 3 = high). Participants could answer “not applicable” where appropriate.

We used the Patient Health Questionnaire-9 (PHQ-9) to assess the presence of major depressive disorder. In total, this instrument includes nine items coded on a scale from 0 to 3 (0 = not at all, 1 = several days, 2 = more than half of the days, 3 = nearly every day). Total scores ranged from 0 to 27, and a higher score suggested the presence of more severe depressive disorder. A cutoff point of five has been previously validated as an appropriate threshold for depression screening (24). Anxiety was measured using the Generalized Anxiety Disorder scale-7 (GAD-7). This instrument is a seven-item scale coded from 0 (none) to 3 (nearly every day). It is based on DSM-IV criteria. Participants were identified as having anxiety if they scored higher than four points.

### Statistical Analysis

The primary outcomes of this study were insomnia (AIS-8 score > 6) and moderate to high degree of fatigue (fatigue scale score ≥ 5). Descriptive data are presented according to the distribution of the variables. Logistic modeling was used to compare participants' contributions to COVID-19 response efforts to their risk of insomnia and fatigue. In step 1, the correlation between participants' roles and the outcomes was tested, controlling for demographic variables (Model 1). In step 2, work-related factors were added, and their potential correlations with the outcomes were considered (Model 2). In step 3, other psychological factors were incorporated into the model, since there were strong correlations between the psychological factors (Model 3). In step 4 (Model 4), social support variables were added to assess how they influenced outcomes. With regard to fatigue, insomnia was also added as an associated factor, since previous studies have documented its correlation with fatigue (9). Finally, interactions between organizational support and participants' roles, work-related factors and mental health statuses were introduced to explore the modifying effects of social support. Modifying factors with a two-tailed *p*-values < 0.05 were considered significant, and are presented. Odds ratios (OR) and 95% confidence intervals (CIs) are reported for all models. All analysis was conducted with SAS 9.4 (SAS Institute Inc., Cary, NC).

## Results

### Characteristics

For this study, a total of 3,619 individuals were recruited to participate in the online survey. After excluding those who were not healthcare workers (62/3,619, 1.7%), a total of 3,557 participants were eligible for subsequent analysis.

Of all eligible participants, 59% (2,099/3,557) worked on the front lines of containment efforts related to the COVID-19 epidemic in China. Participants were predominantly female (2,460/3,557, 69.2%), had bachelor's degrees (1,973/3,557, 55.5%), and were married (2,520/3,557, 70.8%). The majority of the participants were either clinical doctors (1,342/3,557, 37.7%) or nurses (1,333/3,557, 37.5%). Public health practitioners accounted for 8% (285/3,557) of the participants. Consistent with this finding, 85% (3,026/3,557) of participants were working in hospitals, while 230 (6.5%) were working in centers for disease control and prevention (CDCs). Most of the participants reported working over 8 h per day (73.3%). Also, most participants had received a moderate to high degree of social support from organizations and individuals; median scores were 3.0 (2.0, 3.0) and 2.7 (2.0, 3.0), respectively (Table 1).

**Table 1:** Participants' socio-demographic factors, work-related factors, social support, mental health, insomnia and fatigue (*N* %).

	**Non-front line (*N* = 1,458)**	**Front line (*N* = 2,099)**	**Total (*N* = 3,557)**	***P***
**Socio-demographic**				
Sex				<0.001
Male	325 (22.3)	772 (36.8)	1,097 (30.8)	
Female	1,133 (77.7)	1,327 (63.2)	2,460 (69.2)	
Age				<0.001
Mean ± SD	34.5 ± 9.8	37.1 ± 9.1	36.0 ± 9.5	
Min, Max	18.0, 68.0	17.0, 70.0	17.0, 70.0	
Median (Q1, Q3)	33.0 (27.0, 41.0)	36.0 (30.0, 44.0)	35.0 (28.0, 43.0)	
Educational attainment				<0.001
High school or below	64 (4.4)	93 (4.4)	157 (4.4)	
Junior college degree	311 (21.3)	345 (16.4)	656 (18.4)	
Bachelor's degree	733 (50.3)	1240 (59.1)	1,973 (55.5)	
Master's degree	232 (15.9)	316 (15.1)	548 (15.4)	
PhD	118 (8.1)	105 (5.0)	223 (6.3)	
Marital status				<0.001
Single	461 (31.6)	470 (22.4)	931 (26.2)	
Married	962 (66.0)	1,558 (74.2)	2,520 (70.8)	
Divorced/widowed	35 (2.4)	71 (3.4)	106 (3.0)	
Job				<0.001
Clinical doctors	515 (35.3)	827 (39.4)	1342 (37.7)	
Medical lab staff	20 (1.4)	80 (3.8)	100 (2.8)	
Nurses	663 (45.5)	670 (31.9)	1333 (37.5)	
Public health physicians	26 (1.8)	259 (12.3)	285 (8.0)	
Others	234 (16.0)	263 (12.5)	497 (14.0)	
Job title				<0.001
Unemployed	257 (17.6)	145 (6.9)	402 (11.3)	
Entry	568 (39.0)	826 (39.4)	1,394 (39.2)	
Mid-level	389 (26.7)	690 (32.9)	1,079 (30.3)	
Senior	244 (16.7)	438 (20.9)	682 (19.2)	
Employer				<0.001
Hospital	1,348 (92.5)	1,678 (79.9)	3,026 (85.1)	
CDC	7 (0.5)	223 (10.6)	230 (6.5)	
Other	103 (7.1)	198 (9.4)	301 (8.5)	
**Work-related**				
Daily working hours (hours)				<0.001
4~	174 (11.9)	139 (6.6)	313 (8.8)	
6~	269 (18.4)	368 (17.6)	637 (17.9)	
8~	784 (53.8)	905 (43.1)	1,689 (47.5)	
10~	176 (12.1)	351 (16.7)	527 (14.8)	
12~	55 (3.8)	336 (16.0)	391 (11.0)	
Continuous working hours per day (hours)				<0.001
<4	357 (24.5)	219 (10.4)	576 (16.2)	
4~	551 (37.8)	780 (37.2)	1,331 (37.4)	
6~	221 (15.2)	460 (21.9)	681 (19.1)	
8~	329 (22.6)	640 (30.5)	969 (27.2)	
Hours of sleep per day				<0.001
<5	33 (2.3)	91 (4.3)	124 (3.5)	
5~	110 (7.5)	240 (11.4)	350 (9.8)	
6~	468 (32.1)	850 (40.5)	1,318 (37.1)	
7~	586 (40.2)	720 (34.3)	1,306 (36.7)	
8~	261 (17.9)	198 (9.4)	459 (12.9)	
**Social support**				
Organizational support				0.092
Mean ± SD	2.4 ± 0.9	2.5 ± 0.7	2.4 ± 0.8	
Median (Q1, Q3)	3.0 (2.0, 3.0)	3.0 (2.0, 3.0)	3.0 (2.0, 3.0)	
Personal support				0.010
Mean ± SD	2.3 ± 0.8	2.5 ± 0.7	2.4 ± 0.7	
Median (Q1, Q3)	2.7 (2.0, 3.0)	2.7 (2.0, 3.0)	2.7 (2.0, 3.0)	
**Mental health**				
**Depressive status**				
PHQ-9 score				<0.001
Median (Q1, Q3)	4.0 (1.0, 8.0)	4.0 (1.0, 8.0)	4.0 (1.0, 8.0)	
Depression				0.003
Depressed (PHQ-9 score ≤ 4)	812 (55.7)	1,063 (50.6)	1,875 (52.7)	
Not depressed (PHQ-9 score>4)	646 (44.3)	1,036 (49.4)	1,682 (47.3)	
**Anxiety**				
GAD-7 score				<0.001
Median (Q1, Q3)	2.0 (0.0, 6.0)	3.0 (0.0, 6.0)	2.0 (0.0, 6.0)	
Anxiety				0.001
No anxiety (GAD-7 score ≤ 4)	1,001 (68.7)	1,332 (63.5)	2,333 (65.6)	
Anxiety (GAD-7 score >4)	457 (31.3)	767 (36.5)	1,224 (34.4)	
**Insomnia**				
AIS-8 score				<0.001
Mean ± SD	6.6 ± 4.9	8.3 ± 5.0	7.6 ± 5.0	
Median (Q1, Q3)	6.0 (3.0, 9.0)	8.0 (5.0, 12.0)	8.0 (4.0, 11.0)	
Insomnia				<0.001
No Insomnia (AIS-8 score ≤ 6)	744 (51.0)	769 (36.6)	1,513 (42.5)	
Insomnia (AIS-8 score >6)	714 (49.0)	1330 (63.4)	2,044 (57.5)	
**Fatigue**				
Self-rated score				<0.001
Mean ± SD	4.6 ± 2.7	5.8 ± 2.5	5.3 ± 2.6	
Median (Q1, Q3)	5.0 (2.0, 6.0)	6.0 (4.0, 8.0)	6.0 (4.0, 7.0)	
Degree of fatigue				<0.001
0	171 (11.7)	89 (4.2)	260 (7.3)	
1~	203 (13.9)	177 (8.4)	380 (10.7)	
3~	299 (20.5)	318 (15.2)	617 (17.3)	
5~	432 (29.6)	626 (29.8)	1,058 (29.7)	
7~	277 (19.0)	657 (31.3)	934 (26.3)	
9~	76 (5.2)	232 (11.1)	308 (8.7)	

*SD, Standard deviation; Min, Minimum; Max, Maximum; Q1, Lower quartile; Q3, Upper quartile; CDC, Centers for Disease Control and Prevention; PHQ-9, Patient Health Questionnaire-9; GAD-7, Generalized Anxiety Disorder Scale-7; AIS-8, Athens Insomnia Scale-8*.

### Insomnia and Fatigue

The majority (2,044/3,557, 58%) of the participants suffered from insomnia, based on the AIS-8 scale. Front line HCWs were more likely than non-front line HCWs to have insomnia symptoms (1,330/2,099, 63% and 714/1,458, 49%, respectively). Similarly, 72% (1,515/2,099) of front line HCWs reported a moderate to high (score ≥ 5) degree of fatigue. This suggests that this group is more likely to report severe fatigue than non-front line HCWs (785/1,458, 53.8%). Eight point Seven percentage percent of respondents reported feeling burned out or nearly burned out (score ≥ 9: 308/3,557) (Table 1).

### Factors Associated With Insomnia

As presented in Table 2, front line HCWs (OR = 1.62, 95% CI = 1.40–1.87) had higher odds of reporting insomnia symptoms than non-front line HCWs. HCWs who were married (OR = 1.60, 95% CI = 1.31–1.97) or divorced/widowed (OR = 1.84, 95% CI = 1.16–2.91) were found to be at higher risk of insomnia than unmarried HCWs. HCWs who worked in CDC facilities (OR = 2.11, 95% CI = 1.42–3.13) were found to be at higher risk of insomnia than those employed in hospital settings. Younger HCWs (OR = 0.99, 95% CI = 0.97–1.00) also had lower risks of insomnia, as did those who had obtained PhDs (OR = 0.48, 95% CI = 0.30–0.76) relative to those who had only completed middle-or high-school (Table 2, Model 1).

**Table 2:** Logistic regression of factors correlated with insomnia among healthcare workers during the COVID-19 epidemic.

	**Model 1 OR (95% CI)**	**Model 2 OR (95% CI)**	**Model 3 OR (95% CI)**	**Model 4 OR (95% CI)**	**Model 5 OR (95% CI)**
*R*^2^ (Δ*R*^2^)	0.057	0.195 (0.138)	0.494 (0.298)	0.494 (<0.001)	0.495 (0.001)
Chi-square	153.81	404.57	1,070.69	1.24	4.06
*P* value	<0.001	<0.001	<0.001	0.269	0.066
**Step 1: Socio-Demographic**					
**Sex**					
Male	Ref.	Ref.	Ref.	Ref.	Ref.
Female	0.87 (0.73, 1.03)	0.91 (0.76, 1.09)	0.77 (0.62, 0.96)	0.77 (0.62, 0.96)	0.77 (0.62, 0.96)
Age	0.99 (0.97, 1.00)	0.98 (0.96, 0.99)	0.98 (0.96, 0.99)	0.98 (0.96, 0.99)	0.98 (0.96, 0.99)
**Educational attainment**					
High school or below	Ref.	Ref.	Ref.	Ref.	Ref.
Junior college degree	1.04 (0.72, 1.50)	1.03 (0.69, 1.53)	1.00 (0.63, 1.59)	1.01 (0.63, 1.62)	1.01 (0.63, 1.61)
Bachelor's degree	1.00 (0.70, 1.43)	1.06 (0.72, 1.55)	0.92 (0.58, 1.44)	0.93 (0.59, 1.46)	0.93 (0.59, 1.47)
Master's degree	0.82 (0.55, 1.22)	0.90 (0.59, 1.38)	0.67 (0.40, 1.11)	0.67 (0.40, 1.12)	0.68 (0.41, 1.14)
PhD	0.48 (0.30, 0.76)	0.60 (0.37, 0.98)	0.48 (0.27, 0.87)	0.48 (0.27, 0.87)	0.49 (0.27, 0.88)
**Marital status**					
Single	Ref.	Ref.	Ref.	Ref.	Ref.
Married	1.60 (1.31, 1.97)	1.66 (1.33, 2.06)	1.56 (1.20, 2.02)	1.56 (1.21, 2.02)	1.56 (1.20, 2.02)
Divorced/widowed	1.84 (1.16, 2.91)	1.85 (1.13, 3.02)	1.51 (0.83, 2.74)	1.51 (0.83, 2.75)	1.53 (0.84, 2.79)
**Job**					
Clinical doctors	Ref.	Ref.	Ref.	Ref.	Ref.
Medical lab staff	0.76 (0.49, 1.20)	0.93 (0.57, 1.50)	0.75 (0.42, 1.34)	0.75 (0.42, 1.34)	0.75 (0.42, 1.34)
Nurses	0.97 (0.79, 1.20)	1.01 (0.81, 1.26)	1.24 (0.95, 1.61)	1.24 (0.96, 1.61)	1.24 (0.95, 1.60)
Public health physicians	0.83 (0.59, 1.18)	0.74 (0.51, 1.07)	0.74 (0.47, 1.16)	0.74 (0.47, 1.17)	0.74 (0.47, 1.16)
Other	0.75 (0.60, 0.95)	0.81 (0.63, 1.03)	0.86 (0.65, 1.16)	0.87 (0.65, 1.16)	0.86 (0.65, 1.16)
**Job titles**					
Entry	Ref.	Ref.	Ref.	Ref.	Ref.
Mid-level	0.98 (0.76, 1.25)	1.05 (0.81, 1.37)	1.08 (0.79, 1.48)	1.07 (0.78, 1.47)	1.07 (0.78, 1.47)
Senior	1.08 (0.89, 1.31)	1.07 (0.87, 1.31)	1.04 (0.81, 1.34)	1.04 (0.81, 1.33)	1.04 (0.81, 1.33)
None	1.05 (0.80, 1.39)	1.09 (0.81, 1.46)	1.24 (0.87, 1.76)	1.23 (0.87, 1.75)	1.23 (0.86, 1.75)
**Employer**					
Hospital	Ref.	Ref.	Ref.	Ref.	Ref.
CDC	2.11 (1.42, 3.13)	1.54 (1.01, 2.36)	1.42 (0.86, 2.36)	1.43 (0.86, 2.36)	1.42 (0.86, 2.36)
Other	1.13 (0.86, 1.48)	1.15 (0.86, 1.54)	1.10 (0.78, 1.56)	1.09 (0.77, 1.55)	1.11 (0.78, 1.57)
**Type of healthcare workers**					
Non-front line	Ref.	Ref.	Ref.	Ref.	Ref.
Front line	1.62 (1.40, 1.87)	1.33 (1.14, 1.56)	1.60 (1.33, 1.94)	1.62 (1.34, 1.96)	1.89 (0.98, 3.63)
*Step 2: Work-related*					
**Daily working hours**					
4~	··	Ref.	Ref.	Ref.	Ref.
6~	··	0.82 (0.61, 1.11)	0.89 (0.62, 1.27)	0.90 (0.63, 1.29)	0.89 (0.62, 1.28)
8~	··	0.94 (0.72, 1.24)	0.91 (0.65, 1.26)	0.91 (0.66, 1.27)	0.90 (0.65, 1.25)
10~	··	1.78 (1.27, 2.48)	1.46 (0.98, 2.17)	1.46 (0.98, 2.18)	1.44 (0.96, 2.14)
12~	··	1.47 (1.01, 2.14)	1.21 (0.78, 1.90)	1.22 (0.78, 1.91)	1.19 (0.76, 1.86)
**Continuous working hours (hours)**					
<4	··	Ref.	Ref.	Ref.	Ref.
4~	··	1.29 (1.03, 1.61)	1.12 (0.85, 1.46)	1.12 (0.86, 1.46)	1.13 (0.86, 1.47)
6~	··	1.43 (1.11, 1.85)	1.29 (0.95, 1.76)	1.29 (0.95, 1.76)	1.31 (0.96, 1.77)
8~	··	1.68 (1.31, 2.16)	1.44 (1.07, 1.94)	1.44 (1.07, 1.94)	1.45 (1.07, 1.95)
**Daily hours of sleep**					
8~	··	Ref.	Ref.	Ref.	Ref.
<5	··	13.73 (7.38, 25.52)	6.81 (3.33, 13.93)	6.76 (3.30, 13.82)	6.72 (3.29, 13.73)
5~	··	8.54 (6.01, 12.13)	6.95 (4.59, 10.51)	6.95 (4.59, 10.51)	7.04 (4.65, 10.66)
6~	··	3.66 (2.88, 4.67)	3.37 (2.51, 4.51)	3.36 (2.51, 4.51)	3.38 (2.52, 4.53)
7~	··	1.82 (1.44, 2.30)	1.89 (1.42, 2.51)	1.89 (1.42, 2.51)	1.89 (1.42, 2.51)
**Step 3: Mental health**					
**Depression**					
No depression	··	··	Ref.	Ref.	Ref.
Depression	··	··	8.02 (6.51, 9.88)	7.93 (6.44, 9.78)	7.90 (6.40, 9.74)
**Anxiety**					
No anxiety	··	··	Ref.	Ref.	Ref.
Anxiety	··	··	3.16 (2.47, 4.03)	3.13 (2.45, 4.00)	3.13 (2.45, 3.99)
**Step 4: Social support**					
Organizational support	··	··	··	0.96 (0.80, 1.15)	1.20 (0.90, 1.60)
Personal support	··	··	··	0.97 (0.80, 1.17)	0.79 (0.59, 1.07)
**Step 5: Modification effects**					
**Organizational support** **×** **Type of healthcare workers**					
Organizational support × non-front line	··	··	··	··	Ref.
Organizational support × front line	··	··	··	··	0.69 (0.47, 0.99)

*OR, Odds Ratio; CI, Confidence of interval; Ref, Reference; CDC, Centers for Disease Control and Prevention*.

Work-related factors contributed an additional 13.8% of the observed variance in insomnia symptoms. HCWs who worked 10–12 h per day (OR = 1.78, 95% CI = 1.27–2.48) and those who worked 12 h or more per day (OR = 1.47, 95% CI = 1.01–2.14) were at higher risk of insomnia than those who worked 4–6 h per day. Those who worked longer shifts were also more likely to be at risk of insomnia (4~ h vs. <4 h: OR = 1.29, 95% CI = 1.03–1.61; 6~ h vs. <4 h: OR = 1.43, 95% CI = 1.11–1.85; 8~ h vs. <4 h: OR = 1.68, 95% CI = 1.31–2.16). Additionally, lack of sleep was correlated with insomnia. HCWs who slept <5 h were 13.73 times more likely to report insomnia symptoms than those who slept over 8 h (OR = 13.73, 95% CI = 7.38–25.52) (Table 2, Model 2).

Psychological factors explained 29.8% of the variance in reported insomnia symptoms. HCWs who had depressive symptoms (OR = 8.02, 95% CI = 6.51–9.88) and those who had anxiety symptoms (OR = 3.16, 95% CI = 2.47–4.03) had higher risks of insomnia (Table 2, Model 3).

**Figure 1:**
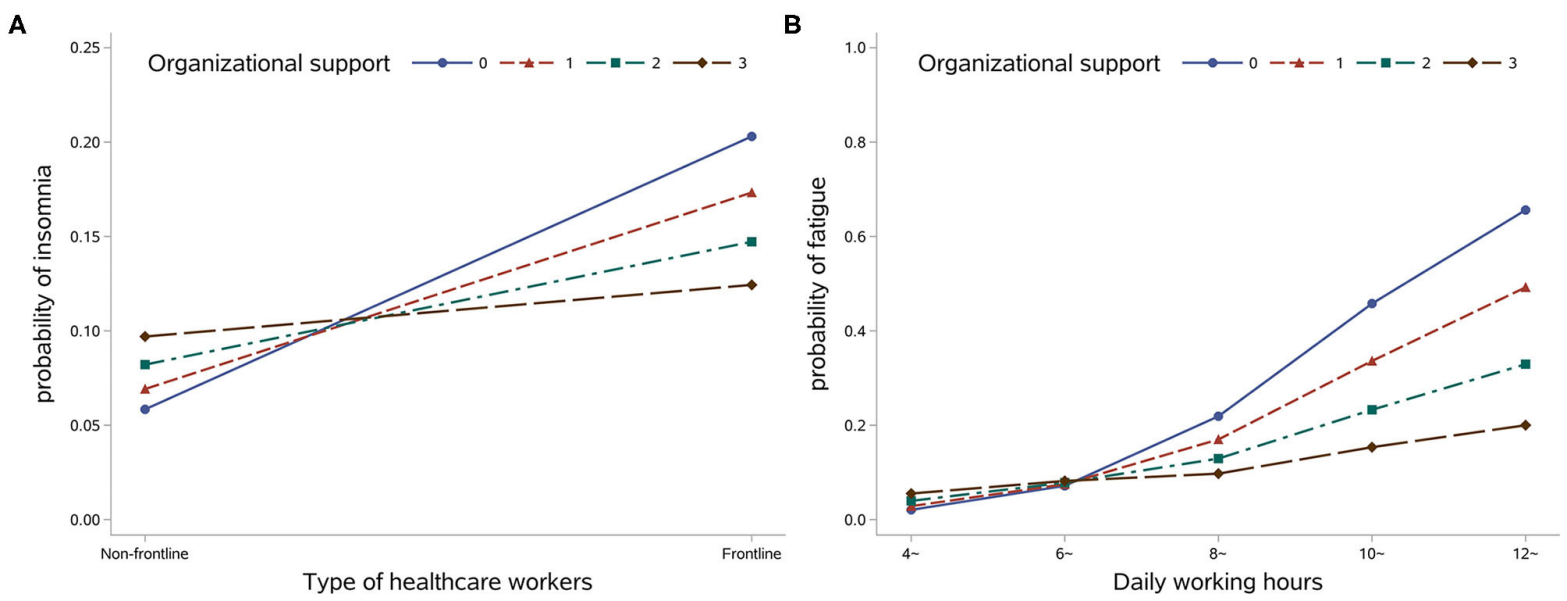
Predicted probability of insomnia among different types of healthcare worker by organizational support level **(A)** and predicted probability of moderate to high degree of fatigue among different types of healthcare worker by organizational support level **(B)**.

Social support only accounted for ~0.1% of the variance in reported insomnia symptoms (Table 2, Model 4). However, organizational support modified the correlation between a HCW's role and their risk of insomnia (OR = 0.69, 95% CI = 0.47–0.99) (Table 2, Model 5). With increasing organizational support, the risk of insomnia among front line HCWs declined, and the difference in insomnia risk between front line and non-front line HCWs decreased even more. Meanwhile, there was no significant influence of organizational support among non-front line HCWs (Figure 1A).

### Factors Associated With Fatigue

The HCWs roles were also associated with fatigue in all models. Front line HCWs (OR = 1.83, 95% CI = 1.58–2.13) were at higher risk of reporting fatigue than non-front line HCWs. Additionally, HCWs who worked in CDCs were more likely to feel fatigued than those who worked in hospitals (OR = 3.59, 95% CI = 2.16–5.97) (Table 3, Model 1).

**Table 3:** Logistic regression of factors correlated with fatigue among healthcare workers during the COVID-19 epidemic.

	**Model 1 OR (95% CI)**	**Model 2 OR (95% CI)**	**Model 3 OR (95% CI)**	**Model 4 OR (95% CI)**	**Model 5 OR (95% CI)**	**Model 6 OR (95% CI)**
*R*^2^ (Δ*R*^2^)	0.085	0.257 (0.173)	0.301 (0.043)	0.326 (0.025)	0.330 (0.004)	0.335 (0.005)
Chi-square	225.663	511.747	141.04	83.609	14.706	16.161
*P-*value	<0.001	<0.001	<0.001	<0.001	<0.001	0.014
**Step 1: Socio-demographic**						
**Sex**						
Male	Ref.	Ref.	Ref.	Ref.	Ref.	Ref.
Female	0.77 (0.64, 0.92)	0.81 (0.67, 0.99)	0.78 (0.64, 0.96)	0.81 (0.66, 0.99)	0.81 (0.66, 1.00)	0.81 (0.66, 1.00)
Age	0.99 (0.98, 1.00)	0.99 (0.97, 1.00)	0.99 (0.97, 1.00)	0.99 (0.98, 1.01)	0.99 (0.98, 1.01)	0.99 (0.98, 1.01)
**Educational attainment**						
High school or below	Ref.	Ref.	Ref.	Ref.	Ref.	Ref.
Junior college degree	1.22 (0.84, 1.76)	1.29 (0.86, 1.94)	1.29 (0.85, 1.96)	1.29 (0.85, 1.97)	1.32 (0.87, 2.02)	1.33 (0.87, 2.04)
Bachelor's degree	1.35 (0.94, 1.92)	1.65 (1.11, 2.45)	1.60 (1.07, 2.39)	1.63 (1.08, 2.45)	1.66 (1.10, 2.50)	1.65 (1.09, 2.49)
Master's degree	1.21 (0.81, 1.82)	1.67 (1.07, 2.62)	1.58 (1.00, 2.51)	1.69 (1.06, 2.70)	1.68 (1.05, 2.69)	1.69 (1.05, 2.70)
PhD	0.82 (0.52, 1.30)	1.40 (0.84, 2.33)	1.40 (0.83, 2.36)	1.56 (0.92, 2.65)	1.55 (0.91, 2.64)	1.52 (0.89, 2.59)
**Marital status**						
Single	Ref.	Ref.	Ref.	Ref.	Ref.	Ref.
Married	1.23 (0.99, 1.52)	1.15 (0.92, 1.45)	1.07 (0.85, 1.35)	1.01 (0.79, 1.27)	1.01 (0.80, 1.28)	1.02 (0.80, 1.29)
Divorced/widowed	1.36 (0.84, 2.20)	1.28 (0.75, 2.17)	1.12 (0.65, 1.94)	1.08 (0.62, 1.87)	1.07 (0.61, 1.86)	1.09 (0.62, 1.90)
**Job**						
Clinical doctors	Ref.	Ref.	Ref.	Ref.	Ref.	Ref.
Medical lab staff	0.80 (0.49, 1.31)	1.08 (0.63, 1.83)	1.02 (0.59, 1.76)	1.10 (0.63, 1.93)	1.09 (0.62, 1.91)	1.10 (0.63, 1.93)
Nurses	1.01 (0.82, 1.25)	1.19 (0.94, 1.50)	1.24 (0.98, 1.58)	1.21 (0.95, 1.54)	1.25 (0.98, 1.59)	1.24 (0.97, 1.58)
Public health practitioners	1.11 (0.76, 1.64)	1.07 (0.70, 1.63)	1.11 (0.72, 1.71)	1.16 (0.75, 1.79)	1.18 (0.76, 1.83)	1.16 (0.75, 1.80)
Other	0.90 (0.71, 1.14)	1.09 (0.84, 1.42)	1.15 (0.88, 1.50)	1.19 (0.91, 1.56)	1.20 (0.91, 1.57)	1.18 (0.90, 1.55)
**Job titles**						
Entry	Ref.	Ref.	Ref.	Ref.	Ref.	Ref.
Mid-level	0.74 (0.58, 0.95)	0.82 (0.63, 1.08)	0.82 (0.62, 1.08)	0.79 (0.60, 1.05)	0.77 (0.58, 1.02)	0.78 (0.58, 1.04)
Senior	1.21 (0.99, 1.49)	1.27 (1.01, 1.58)	1.26 (1.01, 1.58)	1.27 (1.01, 1.60)	1.26 (1.00, 1.59)	1.25 (0.99, 1.57)
None	1.27 (0.95, 1.69)	1.46 (1.06, 1.99)	1.52 (1.10, 2.10)	1.50 (1.08, 2.09)	1.48 (1.06, 2.06)	1.47 (1.06, 2.05)
**Employer**						
Hospital	Ref.	Ref.	Ref.	Ref.	Ref.	Ref.
CDC	3.59 (2.16, 5.97)	2.26 (1.31, 3.90)	2.16 (1.24, 3.77)	2.09 (1.19, 3.68)	2.07 (1.18, 3.64)	2.11 (1.19, 3.73)
Other	1.00 (0.76, 1.32)	1.02 (0.74, 1.39)	0.98 (0.71, 1.36)	0.97 (0.70, 1.35)	0.94 (0.68, 1.32)	0.96 (0.69, 1.34)
**Type of healthcare workers**						
Non-front line	Ref.	Ref.	Ref.	Ref.	Ref.	Ref.
Front line	1.83 (1.58, 2.13)	1.42 (1.20, 1.67)	1.47 (1.25, 1.74)	1.38 (1.16, 1.63)	1.43 (1.20, 1.69)	1.40 (1.18, 1.66)
**Step 2: Work-related**						
**Daily working**						
4~	··	Ref.	Ref.	Ref.	Ref.	Ref.
6~	··	1.58 (1.15, 2.15)	1.69 (1.23, 2.33)	1.74 (1.26, 2.41)	1.78 (1.29, 2.46)	1.83 (0.65, 5.14)
8~	··	2.35 (1.77, 3.12)	2.46 (1.84, 3.29)	2.56 (1.90, 3.44)	2.60 (1.93, 3.49)	6.22 (2.55, 15.17)
10~	··	5.26 (3.66, 7.55)	4.95 (3.42, 7.17)	4.89 (3.36, 7.12)	4.89 (3.36, 7.12)	5.00 (1.61, 15.55)
12~	··	7.26 (4.64, 11.36)	7.22 (4.57, 11.40)	7.36 (4.63, 11.70)	7.45 (4.68, 11.87)	14.38 (2.50, 82.57)
**Continuous working hours**						
<4	··	Ref.	Ref.	Ref.	Ref.	Ref.
4~	··	2.02 (1.61, 2.54)	1.94 (1.53, 2.44)	1.94 (1.53, 2.46)	1.97 (1.55, 2.49)	1.96 (1.54, 2.49)
6~	··	2.23 (1.71, 2.90)	2.16 (1.65, 2.83)	2.13 (1.62, 2.81)	2.13 (1.61, 2.80)	2.13 (1.61, 2.81)
8~	··	2.82 (2.17, 3.67)	2.64 (2.02, 3.46)	2.58 (1.96, 3.39)	2.58 (1.96, 3.39)	2.61 (1.98, 3.44)
**Hours of sleep per day**						
8~	··	Ref.	Ref.	Ref.	Ref.	Ref.
<5	··	7.80 (4.19, 14.52)	5.15 (2.72, 9.77)	4.23 (2.20, 8.12)	4.17 (2.17, 8.00)	4.17 (2.17, 8.04)
5~	··	5.32 (3.71, 7.62)	4.04 (2.80, 5.85)	3.15 (2.16, 4.60)	3.15 (2.16, 4.60)	3.16 (2.16, 4.61)
6~	··	3.05 (2.38, 3.91)	2.66 (2.06, 3.42)	2.25 (1.74, 2.92)	2.26 (1.74, 2.93)	2.25 (1.74, 2.92)
7~	··	1.88 (1.48, 2.39)	1.82 (1.43, 2.32)	1.68 (1.31, 2.15)	1.69 (1.32, 2.16)	1.69 (1.32, 2.16)
**Step 3: Mental health**						
**Depression**						
No depression	··	··	Ref.	Ref.	Ref.	Ref.
Depression	··	··	2.02 (1.65, 2.46)	1.39 (1.11, 1.73)	1.35 (1.08, 1.68)	1.35 (1.08, 1.69)
**Anxiety**						
No Anxiety	··	··	Ref.	Ref.	Ref.	Ref.
Anxiety	··	··	1.52 (1.22, 1.90)	1.30 (1.03, 1.63)	1.27 (1.01, 1.59)	1.27 (1.01, 1.59)
**Step 4: Insomnia**						
**Insomnia**						
No Insomnia	··	··	··	Ref.	Ref.	Ref.
Insomnia	··	··	··	2.45 (2.02, 2.97)	2.44 (2.01, 2.96)	2.42 (2.00, 2.94)
**Step 5: Social support**						
Organizational support	··	··	··	··	0.81 (0.68, 0.96)	1.41 (0.78, 2.53)
Personal support	··	··	··	··	1.01 (0.84, 1.20)	0.69 (0.38, 1.25)
**Step 6: Modification effects**						
**Organizational support** **×** **Daily working hours**						
Organizational support × 4 h	··	··	··	··	··	Ref.
Organizational support × 6 h	··	··	··	··	··	0.74 (0.37, 1.50)
Organizational support × 8 h	··	··	··	··	··	0.52 (0.28, 0.97)
Organizational support × 10 h	··	··	··	··	··	0.43 (0.19, 0.93)
Organizational support × 12 h	··	··	··	··	··	0.36 (0.14, 0.92)

*OR, Odds Ratio; CI, Confidence of interval; Ref, Reference; CDC, Centers for Disease Control and Prevention*.

Work-related factors made the greatest contribution (17.3%) to reported degree of fatigue. Compared with those who worked 4–6 h per day, HCWs who worked more than 12 h per day had the highest odds of reporting fatigue (OR = 7.26, 95% CI = 4.64–11.36). Similarly, compared to those who worked <4 h per day, HCWs who worked 4 continuous hours or more per day were more likely to report a higher degree of fatigue. Compared with those who slept 8 h or more per day, HCWs who slept <8 h per day had higher odds of reporting fatigue (<5 vs. 8~ h: OR = 7.80, 95% CI = 4.19–14.52; 5~ vs. 8~ h: OR = 5.32, 95% CI = 3.71–7.62; 6~ vs. 8~ h: OR = 3.05, 95% CI = 2.38–3.91; 7~ vs. 8~ h: OR = 1.88, 95% CI = 1.48–2.39) (Table 3, Model 2).

Psychological factors accounted for 4.3% of the variance in reported feelings of fatigue. Depressive symptoms (OR = 2.02, 95% CI = 1.65–2.46) and anxiety (OR = 1.52, 95% CI = 1.22–1.90) were considered risk factors for fatigue (Table 3, Model 3). Additionally, insomnia was associated with feelings of fatigue (OR = 2.45, 95% CI = 2.02–2.97), but only explained 2.5% of the total variance (Table 3, Model 4).

Similar to the results regarding insomnia, social support explained an additional 0.4% of the variance in reported feelings of fatigue (Table 3, Model 5). It also modified the correlation between daily working hours and feelings of fatigue (Table 3, Model 6). Organizational support mitigated the positive association between daily work hours and degree of fatigue (Figure 1B).

## Discussion

This study reported that 49 and 63.4% of non-front line and front line HCWs, respectively, experienced insomnia. Moreover, health practitioners employed in CDCs had higher risk of insomnia, and reported a higher degree of fatigue, than clinical doctors. Our results suggest that organizational support modifies the association between HCWs' role and insomnia. It also mitigates the positive correlation between working hours and reported feelings of fatigue.

The percentage of participants reporting symptoms of insomnia in our study exceeded those reported in other studies (34.0~38.4%) (7, 25). This may partly be explained by the different scales [e.g., Insomnia Severity Index (ISI)] for assessing insomnia severity. Several studies have suggested a higher sensitivity when diagnosing insomnia with the AIS-8 than with the ISI. Moreover, AIS-8 has shown superior diagnostic performance in detecting health outcomes associated with insomnia (26, 27). This study reports that during the COVID-19 epidemic, 53.8 and 72.2% of non-front line and front line HCWs, respectively, reported feeling moderate to high degrees of fatigue, and about 10% of participants reported being near exhaustion. These percentages were similar to those obtained in a previous study of self-reported fatigue among HCWs during the SARS outbreak (70.3%) (26). These high percentages for insomnia and feelings of fatigue should be noted as early alerts for additional psychological problems.

Health practitioners working in CDCs, who were critical to curbing the COVID-19 epidemic in China, were at an even higher risk of developing insomnia symptoms than were clinical doctors working in hospitals. During the crisis, HCWs in CDCs were tasked with administrative responsibilities and needed to undertake efforts to contain the disease. They were engaged in work related to disease surveillance, case finding, reporting, close contact tracing, investigation, laboratory testing, disinfecting high-risk public places, health education, training and policy-making (27). Heavy workload and exposure to extreme stress put them at high risk for insomnia and fatigue.

In this study, we found that psychological problems (depression and anxiety) accounted for the largest proportion (29.9%) of variance in reported insomnia symptoms, but only contributed slightly to variance in reported feelings of fatigue (4.4%). Current evidence suggests that the relationship between insomnia and depression can be bidirectional (28). Previous study reported that about 20% of patient with insomnia presented depressive symptoms (29, 30). Insomnia symptoms may have predictive value for subsequent development of depression (31). Other studies reported continued insomnia may become chronic despite successful resolution of depressive symptoms (32). Among those who firstly get insomnia and depression, 29% of patients' insomnia symptoms developed after depressive symptoms (33). Most researchers agreed that mutual effect exist between insomnia and depression (34, 35). Previous studies have reported that fatigue is the most common symptom of insomnia (9, 36). However, we found that insomnia only explained a small proportion (2.4%) of the variance in feelings of fatigue, with these feelings predominantly explained by work-related variables (17.5%). We highlight the need to identify insomnia symptoms in HCWs, and take measures to provide early intervention for psychological problems, considering that a large proportion of the variance in insomnia symptoms can be explained by depression and anxiety. Although the Chinese government has launched a series of measures related to psychological intervention, there remains a need for further studies to evaluate their effects.

A strong association was also shown between work-related factors and both insomnia and fatigue. We found that as daily working hours increased, the risk of insomnia spiked. Similar results have also been reported in other studies conducted during the COVID-19 epidemic. This evidence reveals a close relationship between work overload and insomnia; overload's effect on sleep disturbance can be considerable, especially in working populations (37). Daily working hours was also found to be positively correlated with fatigue, which is consistent with a previous study (38). Moreover, in addition to daily working hours, longer continuous working hours also contributed to insomnia symptoms and feelings of fatigue. During the early stages of the COVID-19 epidemic, HCWs often worked longer each day. Under these circumstances, breaks were crucial to alleviating fatigue (39). In line with previous research, insomnia symptoms and feelings of fatigue were found to be inversely correlated with sleep duration (40). Of note, the odds of insomnia and fatigue spiked when sleeping hours decreased, especially for HCWs who reported sleeping <5 h per day.

At the outset of the COVID-19 epidemic, scarcities of both HCWs and resources made it difficult to divide work shifts between HCWs and to ensure adequate rest. During this stressful situation, organizational support attenuated the positive correlation between working hours and fatigue. This implies that political commitment from the government and broad community participation promote anti-epidemic work (41). The Chinese government has taken several key measures to combat the COVID-19 epidemic, along with implementing additional supporting measures (42). Adequate training, as well as logistical support for HCWs, has been shown to reduce their fears of infection (2, 43). Psychological interventions may also mitigate mental health problems (44). Services provided to HCWs' families could reduce their worries about their families. With a growing number of HCWs participating in the fight against COVID-19, HCWs have gained peer support and had their workloads reduced. All of these measures could mitigate the fatigue symptoms caused by both workload and psychological problems. Organizational support could also attenuate insomnia symptoms. Of note, front line HCWs who faced more stressors were more likely to have insomnia symptoms, and they also received more organizational support. The results of this study suggest that organizational support mitigates insomnia symptoms among front line HCWs.

This study has several limitations. Firstly, participants were not selected as a representative sample of HCWs in China. Secondly, HCWs who were under extreme stress or an extreme workload were less likely to participate in the survey, potentially leading to an underestimation of insomnia and fatigue. Thirdly, questionnaires were shortened to increase the completion rate, meaning that several potential associated factors were not included in this study.

## Conclusion

Front line HCWs in the fight against COVID-19 have reported both insomnia symptoms and feelings of fatigue. Organizational support is negatively correlated with the risk of insomnia symptoms, and mitigates the positive correlation between working hours and degree of fatigue in front line HCWs.

## Data Availability Statement

The original contributions presented in the study are included in the article/supplementary material, further inquiries can be directed to the corresponding author/s.

## Ethics Statement

The studies involving human participants were reviewed and approved by the ethical committee of Sun Yat-sen University [(2020) No. 011]. The patients/participants provided their written informed consent to participate in this study.

## Author Contributions

LL, WC, and XZ designed the study. LL, WC, JY, SL, YY, and FZ collected the data, and SL conducted data analysis. XZ drafted the paper. LL, XZ, SL, JL, YY, and FZ contributed to paper revisions. All authors contributed to the article and approved the submitted version.

## Conflict of Interest

The authors declare that the research was conducted in the absence of any commercial or financial relationships that could be construed as a potential conflict of interest.
